# Vaccination Intention Following Receipt of Vaccine Information Through Interactive Simulation vs Text Among COVID-19 Vaccine–Hesitant Adults During the Omicron Wave in Germany

**DOI:** 10.1001/jamanetworkopen.2022.56208

**Published:** 2023-02-16

**Authors:** Odette Wegwarth, Ulrich Mansmann, Fred Zepp, Dagmar Lühmann, Ralph Hertwig, Martin Scherer

**Affiliations:** 1 Department of Anesthesiology and Intensive Care Medicine, Charité – Universitätsmedizin Berlin, Berlin, Germany; 2 Center for Adaptive Rationality, Max Planck Institute for Human Development, Berlin, Germany; 3 Institute for Medical Information Processing, Biometry, and Epidemiology, Ludwig-Maximilians-Universität München, Munich, Germany; 4 Standing Committee on Vaccination (STIKO), Berlin, Germany; 5 Department of General Practice and Primary Care, University Medical Center Hamburg-Eppendorf, Hamburg, Germany

## Abstract

**Importance:**

Hesitancy toward COVID-19 vaccination is a major factor in stagnating uptake rates and in the risk of health care systems becoming overwhelmed.

**Objective:**

To compare an interactive risk ratio simulation (intervention) with a conventional text-based risk information format (control) and analyze change in participants’ COVID-19 vaccination intention and benefit-to-harm assessment.

**Design, Setting, and Participants:**

Cross-sectional study conducted online with 1255 COVID-19 vaccine–hesitant adult residents of Germany in April and May 2022, surveyed using a probability-based internet panel maintained by respondi, a research and analytics firm. Participants were randomized to 1 of 2 presentations on the benefits and adverse events associated with vaccination.

**Exposure:**

Participants were randomized to a text-based description vs an interactive simulation presenting age-adjusted absolute risks of infection, hospitalization, ICU admission, and death after exposure to coronavirus in vaccinated vs unvaccinated individuals relative to the possible adverse effects as well as additional (population-level) benefits of COVID-19 vaccination.

**Main Outcomes and Measures:**

Absolute change in respondents’ COVID-19 vaccination intention category and benefit-to-harm assessment category.

**Results:**

Participants were 1255 COVID-19 vaccine–hesitant residents of Germany (660 women [52.6%]; mean [SD] age, 43.6 [13.5] years). A total of 651 participants received a text-based description, and 604 participants received an interactive simulation. Relative to the text-based format, the simulation was associated with greater likelihood of positive change in vaccination intentions (19.5% vs 15.3%, respectively; absolute difference, 4.2%; adjusted odds ratio [aOR], 1.45; 95% CI, 1.07-1.96; *P* = .01) and benefit-to-harm assessments (32.6% vs 18.0%; absolute difference, 14.6%; aOR, 2.14; 95% CI, 1.64-2.80; *P* < .001). Both formats were also associated with some negative change. However, the net advantage (positive – negative change) of the interactive simulation over the text-based format was 5.3 percentage points for vaccination intention (9.8% vs 4.5%) and 18.3 percentage points for benefit-to-harm assessment (25.3% vs 7.0%). Positive change in vaccination intention (but not in benefit-to-harm assessment) was associated with some demographic characteristics and attitudes to COVID-19 vaccination; negative changes were not.

**Conclusions and Relevance:**

In this cross-sectional study, vaccine-hesitant adults presented with an interactive risk ratio simulation were more likely to show positive change in COVID-19 vaccination intention and benefit-to-harm assessment than those presented with a conventional text-based information format. These findings suggest that the interactive risk communication format can be an important tool in addressing vaccination hesitancy and fostering public trust.

## Key Points

**Question:**

Can COVID-19 vaccination hesitancy be overcome through effective communication utilizing an interactive simulation?

**Findings:**

In a cross-sectional study of 1255 COVID-19 unvaccinated, vaccine-hesitant residents of Germany, exposure to an interactive simulation presenting the potential benefits and harms of vaccination was associated with greater positive change in people’s intention to receive the COVID-19 vaccine than was exposure to the same information presented in a conventional text-based format.

**Meaning:**

These findings suggest that the interactive risk communication format can be an important tool for health authorities in overcoming vaccine hesitancy and building public trust.

## Teaser

This cross-sectional study evaluates the vaccination intentions of COVID-19 vaccine hesitant adults during the Omicron wave in Germany following receipt of vaccine information through interactive simulation vs text.

## Introduction

COVID-19 vaccines^1,2^ have saved millions of lives since release and remain a key tool in the fight against the pandemic. However, most countries have not reached the vaccine uptake rates needed to relieve pressure on hospitals and intensive care units (ICUs) during peak COVID-19 periods.^3^ In the European Union, for example, vaccination uptake is at 72%, with country-specific rates of twice-vaccinated adults ranging between 50% and 90%. In North America, just 65% of the adult population has been vaccinated twice.^4^ Reduced effectiveness of vaccines in preventing infection with the Omicron variant and milder courses of disease^5^ may have contributed to the belief that vaccination is no longer necessary, especially among vaccine-hesitant individuals. As yet, there is no universally agreed definition of vaccine hesitancy, and the terms “vaccine hesitancy” and “vaccine denial” are often used interchangeably.^6^ But whereas vaccine deniers are unwavering in their resolve not to get vaccinated, vaccine hesitancy is a spectrum. Research shows that vaccine-hesitant individuals are characterized by a high need for information on both benefits and harms, and that they may decide to get vaccinated if that information convinces them.^7^ Given this potential to change minds, the key question is: how can vaccine-hesitant individuals’ needs for balanced risk ratio information best be addressed? Evidence from cognitive and behavioral science suggests that interactive simulations of risk information, which imitate mechanisms by which humans sample risk information naturally (ie, sequentially and experientially), can be more effective in helping people to develop adequate risk perceptions and initiate behavioral change^8,9,10^ than conventional text-based formats.^11,12^ We therefore sought to determine the value of an interactive risk ratio simulation relative to a text-based format in prompting positive change in unvaccinated, vaccine-hesitant respondents’ assessment of the COVID-19 vaccine’s benefit-to-harm ratio and in their intention to get vaccinated during the Omicron wave in Germany.

## Methods

### Study Design

We used the Strengthening the Reporting of Observational Studies in Epidemiology (STROBE) reporting guideline. This study was approved by the institutional review board of the Charité – Universitätsmedizin Berlin. Written informed consent, granted by waiver by the institutional ethics review board of the Charité – Universitätsmedizin, was obtained online from all participants at the outset of the study.

### Participants, Setting, and Survey Design

A cross-sectional national sample of COVID-19 unvaccinated, vaccine-hesitant German residents aged 18 years or older (Table 1) was drawn from a well-established, probability-based internet panel maintained by respondi, a research and analytics firm. Respondents completed the study online between April 1 and May 21, 2022, at the peak of the Omicron wave in Germany. As no official statistics are available on sex, age, ethnicity, and education of COVID-19 vaccine–hesitant individuals in Germany, a quota sampling method could not be employed. To ensure eligibility, respondents answered 2 screening questions concerning (1) their current vaccination status (“Have you been vaccinated against COVID-19 yet? [yes/no]”) and (2) their intention to get vaccinated against COVID-19 in the foreseeable future (5-point scale; response options: “I will definitely get the COVID-19 vaccination,” “I will probably get the COVID-19 vaccination,” “I am unsure if I will get the COVID-19 vaccination,” “I will probably not get the COVID-19 vaccination,” and “I will definitely not get the COVID-19 vaccination”). Only respondents who had not yet received a COVID-19 vaccination and expressed indecisiveness (“probably,” “unsure,” or “probably not”) about their vaccination intention were able to access the questionnaire; those who were already vaccinated or decided were excluded from participation. Because the German federal government implemented mandatory COVID-19 vaccination for health care professionals from March 16, 2022—a measure that sparked widespread controversy—we next asked whether respondents were employed in the health care sector to assess the potential influence of mandatory vaccination. We further assessed satisfaction with the government’s COVID-19 containment strategies and asked respondents for their subjective assessment of the benefit-to-harm ratio of COVID-19 vaccination, measured with the following 5-point response scale: “The benefits of the COVID-19 vaccination clearly outweigh the harms,” “The benefits of the COVID-19 vaccination somewhat outweigh the harms,” “Benefits and harms of the COVID-19 vaccination are balanced,” “The harms of the COVID-19 vaccination somewhat outweigh the benefits,” “The harms of the COVID-19 vaccination clearly outweigh the benefits.” Finally, respondents were asked a series of questions about their general attitudes to COVID-19 vaccination (eAppendix 1 in Supplement 1) before being randomly assigned to 1 of 2 treatment conditions: a text-based description or an interactive simulation of the benefit-to-harm ratio of COVID-19 vaccination (eAppendix 2 in Supplement 1). The text-based description was the control condition as it reflects the conventional format used to inform people about matters of health; the interactive simulation was the intervention condition. The presentation of risk information in the 2 conditions differed by design (static vs interactive), but the content was identical and followed guidelines for evidence-based health information.^13^ In both interventions, respondents were informed about age-adjusted absolute risks of infection, hospitalization, ICU admission, and death after exposure to COVID-19 in 100 000 vaccinated vs 100 000 unvaccinated individuals relative to the possible adverse effects of vaccination (eg, myocarditis in men ≤35 years). Information was provided on 4 age groups (18–34 years, 35–59 years, 60–79 years, ≥80 years) due to considerable differences in the respective risks (eTable 1 in Supplement 1). Respondents were further informed about the benefits of vaccination for the population as a whole (herd) in terms of preventing infections and deaths. Estimations were based on official statistics from the Robert Koch Institute,^14^ technical briefings of the UK Health Security Agency,^15^ and data from Clalit Health Services (Israel)^16^ (eTable1 in Supplement 1). After exposure to 1 of the 2 risk communication formats, respondents’ vaccination intentions and benefit-to-harm assessments were again measured using the same 5-point scales.

**Table 1.** Summary of Demographic Characteristics by Group^a^

Characteristic	No. (%)		*P* value^b^
	Interactive risk ratio simulation (n = 604)	Text-based risk ratio information (n = 651)	
Age, y			
18-34	178 (29.5)	191 (29.3)	.63
35-59	346 (57.3)	385 (59.1)	
≥60	80 (13.2)	75 (11.5)	
Sex			
Female	327 (54.1)	333 (48.8)	.32
Male	277 (45.9)	318 (51.2)	
Education			
No qualifications/no information^b^	9 (1.5)	10 (1.5)	.04
Basic	53 (8.8)	50 (7.7)	
Intermediate	180 (29.8)	194 (29.8)	
Qualified for higher education	182 (30.1)	155 (23.8)	
Higher education degree	180 (29.8)	242 (37.2)	
Health care professional			
No	548 (90.7)	599 (92.0)	.48
Yes	56 (9.3)	52 (8.0)	

^a^ Percentages are rounded and may not add up to 100.

^b^ Significance at the 2-sided 5% level.

### Primary End Point Measures

Primary end points were the absolute proportions of respondents showing positive change in vaccination intention category and benefit-to-harm assessment category. Positive change could range from a change of 1 category (eg, from “unsure” to “probably”) to 3 categories (from “probably not” to “definitely”) for vaccination intentions and to 4 categories (from “The harms … clearly outweigh the benefits” to “The benefits … clearly outweigh the harms”) for benefit-to-harm assessment. As mentioned earlier, those stating that they would “definitely” or “definitely not” get vaccinated at baseline were excluded from the sample of vaccine-hesitant respondents, which is why the maximum potential change of the 2 end points differed. Secondary end points were negative changes from baseline to postintervention and associations of primary end points with demographic characteristics and attitudes to COVID-19 vaccination.

### Statistical Analyses

The online questionnaire did not allow for item nonresponse; thus, all questionnaires were fully complete. Mann-Whitney–Wilcoxon rank testing was used to test whether the 2 information formats were associated with different likelihoods of positive (and negative) change in categories of vaccination intention and benefit-to-harm assessment (absolute percentages of respondents) from baseline to postintervention. Log-linear analyses were performed to investigate associations of likelihood of positive (and negative) change with assessment of the government’s COVID-19 containment strategies, attitudes to COVID-19 vaccination, and demographic characteristics. Log-linear analyses were adjusted for nonbalanced variables between groups at baseline (education, benefit-to-harm assessment, assessment of governmental containment strategies, and vaccine-related concern about long-term adverse effects due to novelty). Reported adjusted odds ratios (aOR) reflect the likelihood of positive or negative change (across 1 or more) categories from baseline to postintervention. Percentage values are absolute percentage points. *P* values <.05 (2-sided) were considered statistically significant. Analyses were performed with R version 4.2.0 (R Project for Statistical Computing).

## Results

Of the 1255 vaccine-hesitant participants who completed the survey (mean [SD] age, 43.6 [13.5] years), 87.6% were between 18 and 59 years old (1100 participants), 52.6% were female (660 participants), and 60.5% reported having qualified for or completed higher education (759 participants) (Table 1). The vaccine-hesitant sample was younger and more educated than the general German population, in line with the findings of other national COVID-19 surveys.^17^ At baseline, the control group (651 participants) and the intervention group (604 participants) did not differ with respect to vaccination intention (*P* = .31; odds ratio [OR], 1.11; 95% CI, 0.89-1.39), but they did differ in their benefit-to-harm assessment, with respondents in the intervention group being more negative in their assessments: 55.0% in the interactive simulation group (vs 47.8% in the text-based format group) indicated that the vaccination’s harms “clearly” or “somewhat” outweighed its benefits (OR, 1.34; 95% CI, 1.08-1.68; *P* = .01). In addition, respondents in the intervention group were less educated (OR, 0.72; 95% CI, 0.57-0.91; *P* = .04) (Table 1), less satisfied with the government’s COVID-19 containment strategies (OR, 1.52; 95% CI, 1.20-1.94; *P* < .001), and more concerned about potential long-term adverse effects of the novel vaccine (OR, 1.18; 95% CI, 1.04-1.39; *P* = .03) than were those in control group (Table 2).

**Table 2.** Attitudes to COVID-19 Measures and Vaccination: Differences Between Control and Intervention Group, and Association With Positive Change in Vaccination Intention and Benefit-to-Harm-Assessment^a^

Attitude	Interactive risk ratio simulation (n = 604)	Text-based risk ratio information (n = 651)	*P* value for difference between groups^b^	Positive change in vaccination intention across sample, adjusted odds ratio (95% CI)	*P* value^b^	Positive change in benefit-to-harm assessment across sample, adjusted odds ratio (95% CI)	*P* value^b^
Assessment of governmental containment strategies							
Insufficient (dissatisfied with strategy)^c^	135 (22.4)	132 (20.3)	<.001	1 [Reference]	<.001	1 [Reference]	.61
Excessive (dissatisfied with strategy)	308 (51.0)	287 (44.1)		1.23 (0.80-1.88)		1.19 (0.84-1.67)	
Appropriate (satisfied with strategy)	161 (26.7)	232 (35.6)		1.77 (1.28-2.46)		0.94 (0.70-1.27)	
I worry about the potential adverse effects of COVID-19 vaccination							
Yes^c^	519 (85.9)	549 (84.3)	.48	1 [Reference]	.01	1 [Reference]	.14
No	85 (14.1)	102 (15.7)		1.67 (1.14-2.44)		0.75 (0.51-1.11)	
The COVID-19 vaccine is so novel that we don't yet properly understand the long-term adverse effects							
Yes^c^	499 (82.6)	505 (77.6)	.03	1 [Reference]	<.01	1 [Reference]	.87
No	105 (17.4)	146 (22.4)		1.72 (1.22 to 2.41)		0.97 (0.70-1.35)	
In my view the coronavirus is not bad enough to require a vaccination							
Yes^c^	215 (35.6)	247 (37.9)	.42	1 [Reference]	.07	1 [Reference]	.32
No	389 (64.4)	404 (62.1)		1.33 (0.97-1.82)		1.15 (0.87-1.51)	
I my view vaccination does not provide reliable protection against the virus							
Yes^c^	455 (75.3)	466 (71.6)	.15	1 [Reference]	<.001	1 [Reference]	.52
No	149 (24.7)	185 (28.4)		2.09 (1.54-2.86)		1.12 (0.84-1.51)	
I generally avoid vaccinations							
Yes^c^	161 (26.7)	179 (27.5)	.79	1 [Reference]	.67	1 [Reference]	.68
No	443 (73.3)	472 (72.5)		0.93 (0.67-1.29)		0.94 (0.70-1.26)	
It is too complicated for me to get access to the vaccine (eg, the vaccination center is too far away)							
Yes^c^	107 (17.7)	142 (21.8)	.08	1 [Reference]	.05	1 [Reference]	.86
No	497 (82.3)	509 (78.2)		0.70 (0.49-0.99)		0.97 (0.70-1.35)	
In my view the regulatory agencies have not yet disclosed the whole truth about the adverse effects of vaccination							
Yes^c^	438 (72.5)	475 (73.0)	.91	1 [Reference]	.05	1 [Reference]	.81
No	166 (27.5)	176 (27.0)		1.38 (1.01-1.90)		0.96 (0.72-1.29)	

^a^ Percentages are rounded and may not add up to 100.

^b^ Significance at the 2-sided 5% level.

^c^ Reference category for the log-linear analysis.

After intervention and relative to the text-based format, the interactive simulation was associated with greater likelihood of positive change in respondents’ intentions to receive the COVID-19 vaccination (19.5% intervention vs 15.3% control; absolute difference, 4.2%; adjusted OR [aOR], 1.45; 95% CI, 1.07-1.96; *P* = .01) and benefit-to-harm assessment (32.6% intervention vs 18.0% control; absolute difference, 14.6%; aOR, 2.14; 95% CI, 1.64-2.80; *P* < .001) (Figure). We also observed some negative change. However, the proportions of respondents who showed a decline were comparable across the 2 groups for both vaccination intention (9.7% intervention vs 10.8% control; aOR, 0.85; 95% CI, 0.59-1.23; *P* = .39) and benefit-to-harm assessment (7.3% intervention vs 11.0% control; aOR, 0.70; 95% CI, 0.47-1.05; *P* = .08). The net advantage (percentage of absolute positive change – percentage of absolute negative change) of the interactive simulation over the text-based format was 5.3 percentage points for vaccination intention (9.8% vs 4.5%) and 18.3 percentage points for benefit-to-harm assessment (25.3% vs 7.0%).

**Figure.**
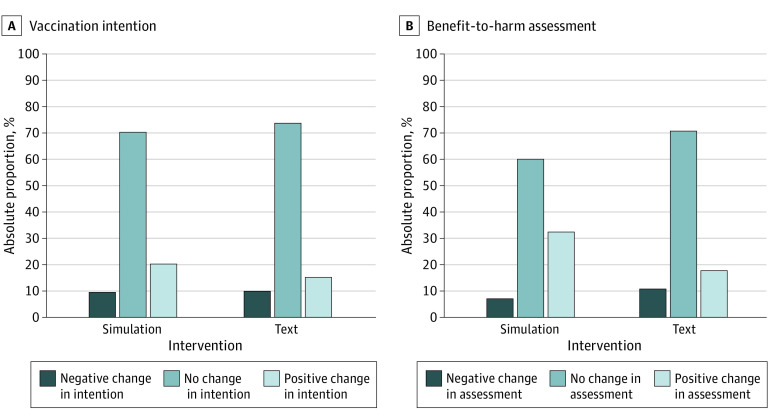
Change in Response Category From Baseline to Postintervention in COVID-19 Vaccine–Hesitant Respondents’ Vaccination Intention and Benefit-to-Harm Assessment

Adjusted multivariable analyses showed that being male (aOR, 1.37; 95% CI, 1.02-1.85; *P* = .04), having qualified for or attended higher educated (aOR, 1.86; 95% CI, 1.35-2.57; *P* < .001), and being satisfied with the government’s COVID-19 containment strategies (aOR, 1.77; 95% CI, 1.28-2.46; *P* < .001) was associated with an increased likelihood of positive change in vaccination intention in both conditions, but not with an increased likelihood of positive change in benefit-to-harm assessment. Likewise, some vaccine-related attitudes were associated with positive change in vaccination intention but not in benefit-to-harm assessment (Table 2). Among the more unexpected findings was that avoidance of vaccination in general did not undermine the likelihood of positive change in COVID-19 vaccination intention (aOR, 0.93; 95% CI, 0.67-1.29; *P* = .67). In contrast, the adjusted multivariable analyses found no associations of demographic characteristics and attitudes to COVID-19 measures and vaccination with negative changes in vaccination intention or benefit-to-harm assessment.

Across the sample, respondents who were health care professionals and thus subject to mandatory vaccination were nearly twice as likely to show positive change in benefit-to-harm assessment after intervention (OR, 1.53; 95% CI, 1.02-2.36; *P* = .04) but only half as likely to show positive change in vaccination intention (OR, 0.47, 95% CI: 0.25-0.90; *P* = .04) than all other respondents.

## Discussion

The COVID-19 pandemic has highlighted the vital role of vaccination in preventing life-threatening diseases and the overload of health care systems. Yet the uptake of COVID-19 vaccination—and even more so of booster shots—remains insufficient in many countries. Against this backdrop, every single person who overcomes vaccine hesitancy counts. In our cross-sectional study of 1255 COVID-19 unvaccinated, vaccine-hesitant residents of Germany, we found that conventional text-based information formats may be less helpful in reaching vaccine-hesitant people than interactive risk ratio simulations; a significantly higher proportion of vaccine-hesitant individuals exposed to the interactive format showed positive change in their intention to receive the COVID-19 vaccine and assessment of the vaccination’s benefits and harms. Considering that more respondents in the interactive simulation group initially thought that the harms of the vaccination outweighed its benefits and expressed dissatisfaction with the government’s COVID-19 containment strategies—a factor negatively associated with the likelihood of a positive intentional change—the study’s results may even underestimate what interactive risk simulations can potentially contribute to fighting vaccination hesitancy.

The outcomes of behavioral interventions in increasing the understanding and/or uptake of vaccination have been tested previously, also in the context of COVID-19 vaccine.^9,12,18^ However, these studies did not limit the analysis to declared vaccine-hesitant respondents or test an experience-based format (interactive risk simulation) against a conventional text-based risk format. To the best of our knowledge, this is the first study to investigate the outcome of 2 behavioral interventions that tap into different cognitive mechanisms in a sample of unvaccinated, vaccine-hesitant adults.

We also observed some negative changes in vaccination intention and benefit-to-risk assessment in both groups. Because reasons for change were not assessed, we can only speculate as to why this occurred. Evidence from a large study with representative samples of US residents and Danes (with more than 13,000 participants)^19^ showed that disclosing information on the potential harms of COVID-19 vaccination can increase vaccine hesitancy. Indeed, concern about jeopardizing public vaccine acceptance is likely what prevents governments, including the German government, from transparently communicating information about potential harms.^19,20^ Yet Petersen and colleagues^19^ found that transparency about the potential harms of COVID-19 vaccines considerably increased people’s trust in health authorities and helped to contain the spread of conspiracy beliefs; they consequently warned against withholding information. In the present study, we deliberately reported potential harms, in accordance with evidence-based transparent reporting in health care. It seems likely that this was the first time that most respondents saw the full picture of the benefit-to-harm ratio. Thus, while transparent communication of potential harms may have been associated with increased vaccine hesitancy in some respondents in the short term, it may serve to build and maintain public trust in the long term.^21,22,23^

Finally, our results suggest that those who have not yet been convinced to get vaccinated by current communication campaigns should probably not be required to do so by law. Whereas respondents from the health care sector were particularly likely to show positive change in their assessment of the vaccination’s benefits and harms after intervention, they were particularly unlikely to show positive change in vaccination intention.

### Limitations

Our study has several limitations. First, the generalizability of results may be limited by our sample, which consisted of residents of Germany only. Second, due to the cross-sectional design, it remains unclear how the behavioral interventions may affect respondents’ behavior in the long term, including their future decisions on COVID-19 vaccination. Third, in keeping the questionnaire short to motivate respondents to complete it, we did not include questions on why respondents did or did not change their intentions and benefit-to-harm assessments, which is why we cannot shed light on the reasoning behind these changes. Fourth, we cannot exclude the likelihood that the vaccine-hesitant respondents who completed the survey were more motivated than the vaccine-hesitant population in general, which may again limit generalizability of results. Fifth, some respondents may have felt they needed to change their initial response after being exposed to either intervention; however, if such a demand effect had played a role it would have affected both groups. Sixth, we cannot rule out the existence of nonrespondent bias.

## Conclusions

Health authorities around the world face an enormous challenge in increasing the uptake of COVID-19 (booster) vaccines. Research from cognitive and behavioral science can help to analyze the situation and design interventions that, ideally, boost trust, understanding, and intention. Our cross-sectional study suggests that vaccine-hesitant people might benefit more from interactive risk simulations than from text-based formats. There is no guarantee that this intervention will work in all contexts and under all conditions. More work on the underlying mechanisms is needed in order for the full potential of such simulations to be understood and harnessed. In future pandemics, interactive simulations that transparently communicate the benefits and harms of vaccination (and uncertainty where it exists) can be an important new instrument in the toolbox of health authorities seeking to overcome vaccine hesitancy and build public trust.

## Notes

Supplement 1. **eAppendix 1. **Questionnaire **eAppendix 2.** Interventions **eTable 1.** Risk Ratio for Vaccine-Related Outcomes

Supplement 2.
Data Sharing Statement
