# Supplementary material for: Vaccination Intention Following Receipt of Vaccine Information Through Interactive Simulation vs Text Among COVID-19 Vaccine–Hesitant Adults During the Omicron Wave in Germany
Source: JAMA Netw Open. 2023 Feb 16;6(2):e2256208. doi: 10.1001/jamanetworkopen.2022.56208 (PMC9936332; doi:10.1001/jamanetworkopen.2022.56208)
Supplement: Supplement 1. — eAppendix 1. Questionnaire eAppendix 2. Interventions eTable 1. Risk Ratio for Vaccine-Related Outcomes [file jamanetwopen-e2256208-s001.pdf]

Supplemental Online Content

Wegwarth O, Mansmann U, Zepp F, Lühmann D, Hertwig R, Scherer M. Vaccination intention following receipt of vaccine information through interactive simulation vs text among COVID-19 vaccine–hesitant adults during the Omicron wave in Germany. *JAMA Netw Open*. 2023;6(2):e2256208. doi:10.1001/jamanetworkopen.2022.56208

eAppendix 1. Questionnaire

eAppendix 2. Interventions

eTable 1. Risk Ratio for Vaccine-Related Outcomes

This supplemental material has been provided by the authors to give readers additional information about their work.

© 2023 Wegwarth O et al. *JAMA Network Open.*

eAppendix 1. Questionnaire (translated to English from German version)

[Pre-Intervention Questionnaire]

1) Have you been vaccinated against COVID-19 yet?

- Yes. → [screen out]
- No. → [move on]

2) Which of the following statements about the COVID-19 vaccination most accurately describes your current intention to receive the COVID-19 vaccination in the nearer future?

- I will definitely get the COVID-19 vaccination in the nearer future in the nearer future. → [decided | screen out].
- I will probably get the COVID-19 vaccination in the nearer future. → [undecided | move on]
- I am unsure if I will get the COVID-19 vaccination in the nearer future. → [undecided | move on]
- I will probably not get the COVID-19 vaccination in the nearer future. → [undecided | move on]
- I will definitely not get the COVID-19 vaccination in the nearer future. → [decided | screen out]

3) Do you work in a profession of the health-care sector for which the government released a compulsory COVID-19 vaccination from March 2022 on?

- Yes
- No

4) When you think of the governmental corona-related containment measures, how satisfied do you feel with these?

- I perceived the containment measures as completely appropriate. [for analysis: coded to "appropriate"]
- I perceived the containment measures as partly appropriate. [for analysis: coded to "appropriate"]
- I perceived the containment measures as partly exaggerated. [for analysis: coded to "exaggerated"]
- I perceived the containment measures as completely exaggerated. [for analysis: coded to "exaggerated"]
- I perceived the containment measures as partly insufficient. [for analysis: coded to "insufficient"]
- I perceived the containment measures as completely insufficient. [for analysis: coded to "insufficient"]
- I perceived the containment measures sometimes as excessive and sometimes as insufficient. [for analysis: coded to "insufficient"]

© 2023 Wegwarth O et al. *JAMA Network Open.*

5. You have not been vaccinated against COVID-19 yet. Please go through the lists of reasons and indicate which of the reasons has been decisive for your decision so far.

- I worry about the potential side effects of a COVID-19 vaccination.
- The COVID-19 vaccine is so novel that I'm afraid we don't yet adequately understand the long-term side effects.
- I think that the coronavirus is not so bad that I need to be vaccinated.
- I think that the vaccine does not provide reliable protection against the coronavirus.
- I avoid vaccinations in general.
- It has been too stressful to get uncomplicated access to the vaccination (e.g., vaccination center is far away; lack of clarity about where or how to get vaccination in a timely manner, etc.)
- I think that the regulatory agencies have not yet disclosed the whole truth about the vaccine's side effects.

6) In your view, which statement best described the ratio between the COVID-19 vaccination’s benefits and harms?

- The benefits of the COVID-19 vaccination clearly outweigh its potential harms.
- The benefits of the COVID-19 vaccination somewhat outweigh its potential harms.
- The benefits and harms of the COVID-19 vaccination are balanced.
- The harms of the COVID-19 vaccination somewhat outweigh its benefits.
- The harms of the COVID-19 vaccination clearly outweigh its benefits.

[*Post-Intervention Questionnaire*]

1. After you have seen the information about the vaccination’s benefits and harms, in your view, which statement best described the ratio between the COVID-19 vaccination’s benefits and harms?

- The benefits of the COVID-19 vaccination clearly outweigh its potential harms.
- The benefits of the COVID-19 vaccination somewhat outweigh its potential harms.
- The benefits and harms of the COVID-19 vaccination are balanced.
- The harms of the COVID-19 vaccination somewhat outweigh its benefits.
- The harms of the COVID-19 vaccination clearly outweigh its benefits.

2. After you have seen the information about the vaccination’s benefits and harms, which of the following statements most accurately describes your current intention to receive the COVID-19 vaccination in the nearer future?

- I will definitely get vaccinated against COVID-19 in the nearer future.
- I will probably get vaccinated against COVID-19 in the nearer future.
- I am undecided whether to get vaccinated against COVID-19 in the nearer future.
- I will probably not get vaccinated against COVID-19 in the nearer future.
- I will definitely not get vaccinated against COVID-19 in the nearer future.

© 2023 Wegwarth O et al. *JAMA Network Open.*

eAppendix 2. Interventions

Introduction for Both Interventions

In March 2020, the World Health Organization declared COVID-19 a global pandemic. Since then, the highly contagious coronavirus (SARS-CoV-2), with its in part profound consequences for health and the healthcare system, has greatly changed all our lives and social interaction. The extent to which the coronavirus affects our lives also depends on the respective variant. Compared to the delta variant, the current omicron variant is more infectious but does cause less severe courses. What future corona-associated variants may bring cannot be reliably estimated at this time and needs to be determined.

Since the beginning of 2021, numerous vaccines have been approved to protect us and others from severe courses of a corona infection. For instance, the mRNA vaccines from BioNTech and Moderna, which are widely used in Germany, or, most recently, the inactivated vaccine from Novavax. All these vaccines are similarly effective, so the information on benefits applies equally to these vaccines. However, we currently do not have sufficient information on the potential risks of Novavax's inactivated vaccine, which is why our following information on risks only applies to BioNTech and Moderna.

© 2023 Wegwarth O et al. *JAMA Network Open.*

Text-based intervention

Personal risk

Even though the currently predominant omicron variant poses less of a health risk than the delta variant of the coronavirus, the following still applies, especially in view of the coming fall: Getting a COVID-19 vaccine protects your health. By getting vaccinated, you lower your personal risk of becoming severely ill with COVID-19, being hospitalized or admitted to the intensive care unit, or dying from COVID-19.

With respect to the Omicron variant, vaccination reduces your risk of infection still by approximately 60%. In the event that you are infected with COVID-19, the vaccination reduces your age-specific risk of being hospitalized due to your corona infection from about [***18-34y***: 0.6% to 0.2%; ***35-59y***: 1.8% to 0.7%; ***60-79y***: 6% to 3.6%; ***>79y***: 16% to 6.4%], your risk of being admitted to the ICU from about [***18-34y***: 0.04% to 0.01%; ***35-59y***: 0.11% to 0.02%; ***60-79y***: from 1% to 0.2%; ***>79y***: 2% to 0.3%], and your risk of dying from coronavirus infection from about [***18-34y***: 0.001% to 0.0002%; ***35-59y***: 0. 03% to 0.005%; ***60-79y***: 1% to 0.2%; ***>79y***: 3% to 0.5%].

While the vaccine can have direct benefits on your health, it may also come with the potential of relevant side effects. “Relevant” means in this context that the side effect requires medical attention (e.g., hives, heart muscle inflammation/myocarditis). Short-term side effects such as fatigue, pain at the injection site, or some fever are therefore not included. Observed relevant side effects sum up to a maximum of 0.01% of cases. In younger men (≤ 35 years), an additional increased risk of about 0.01% for myocarditis or pericarditis was observed within the first two weeks after vaccination.

Two other fears are often named in connection with mRNA vaccines: the fear of long-term effects and the fear of a change in one's own DNA. In this case, the expression “long-term effects“ is mistakenly understood to be a side effect that is first experienced months or years after the vaccination. In the context of vaccinations, however, such side effects do not exist. It is true that in the past, some vaccines (e.g., the smallpox vaccination) caused, in very rare cases, physical reactions that had a long-term effect on an individual’s health (= long-term effect). However, such a reaction only occurred promptly after vaccination. The mRNA-based COVID-19 vaccine has been administered more than 10 billion times in the last 14 months. No side effects have been observed that first arose months after vaccination.

The term “mRNA“ also leads to the erroneous belief that the vaccine penetrates our human genetic material – DNA. This is also incorrect. Rather, mRNA acts as a messenger molecule that fools our body into thinking it is under viral attack. This triggers an immune response in the body. This immune response enables future protection against the virus. Only 50 hours after vaccination, the mRNA has already been broken down by our body.

© 2023 Wegwarth O et al. *JAMA Network Open.*

Because your risk is highly dependent on current pandemic events (e.g., infectiousness of the virus, your contact with others), all risk information should be considered as estimates.

SOCIETAL RISK

A COVID-19 vaccination protects not only yourself, but also those around you. With your vaccination against COVID-19 you contribute to what is known as "herd immunity." If a sufficiently large part of the population (herd) is immunized against the disease, transmission of the virus from person to person becomes increasingly unlikely. On the one hand, you will protect elderly and immunocompromised people in particular from the sometimes-serious health risks of infection, which also exist with the current, milder omicron variant. On the other hand, you are already helping to build sufficient herd immunity for the fall months, which will protect healthcare workers from potential overload resulting from new virus variants.

Under the omicron variant of the coronavirus, it is currently the case that one infected person can infect between 12 to 16 other people. Even though the omicron variant takes a less harmful course than the delta variant, an infection still means for elderly and immunocompromised people that up to 16% are hospitalized, up to 2% are transferred to an intensive care unit due to the severity of the infection, and up to 3% of elderly and immunocompromised people die from the viral disease. If about 90% of all people in Germany were vaccinated, these figures could be reduced by up to 85%.

Since the actual risk is always highly dependent on the actual pandemic situation, the values are to be understood as estimates.

© 2023 Wegwarth O et al. *JAMA Network Open.*

Interactive Risk Simulation (Text) (full version: https://iwill-simulation.mpib-berlin.mpg.de/start)

Personal risk

Even though the currently predominant omicron variant poses less of a health risk than the delta variant of the coronavirus, the following still applies, especially in view of the coming fall: Getting a COVID-19 vaccine protects your health. By getting vaccinated, you lower your personal risk of becoming severely ill with COVID-19, being hospitalized or admitted to the intensive care unit, or dying from COVID-19.

While the vaccine can have direct benefits on your health, it may also come with side effects. The simulation shows the probability of experiencing relevant side effects of the vaccine (e.g., hives, heart muscle inflammation/myocarditis). “Relevant” means in this context that the side effect requires medical attention. Short-term side effects such as fatigue, pain at the injection site, or fever are therefore not included.

In the simulation below, we show how these risks apply to your age group and change depending on vaccination status.

Two other fears are often named in connection with mRNA vaccines: the fear of long-term effects and the fear of a change in one's own DNA. In this case, the expression “long-term effects“ is mistakenly understood to be a side effect that is first experienced months or years after the vaccination. In the context of vaccinations, however, such side effects do not exist. It is true that in the past, some vaccines (e.g., the smallpox vaccination) caused, in very rare cases, physical reactions that had a long-term effect on an individual’s health (= long-term effect). However, such a reaction only occurred promptly after vaccination. The mRNA-based COVID-19 vaccine has been administered more than 10 billion times in the last 14 months. No side effects have been observed that first arose months after vaccination.

The term “mRNA“ also leads to the erroneous belief that the vaccine penetrates our human genetic material – DNA. This is also incorrect. Rather, mRNA acts as a messenger molecule that fools our body into thinking it is under viral attack. This triggers an immune response in the body. This immune response enables future protection against the virus. Only 50 hours after vaccination, the mRNA has already been broken down by our body.

Because your risk is highly dependent on current pandemic events (e.g., infectiousness of the virus, your contact with others), all risk information shown should be considered as estimates.

[interactive risk simulation/age-adjusted risk for incidence, hospital, ICU, death]

© 2023 Wegwarth O et al. *JAMA Network Open.*

SOCIETAL RISK

A COVID-19 vaccination protects not only yourself, but also those around you. With your vaccination against COVID-19 you contribute to what is known as “herd immunity.“ If a sufficiently large part of the population (herd) is immunized against the disease, transmission of the virus from person to person becomes increasingly unlikely. On the one hand, you will protect elderly and immunocompromised people in particular from the sometimes-serious health risks of infection, which also exist with the current, milder omicron variant. On the other hand, you are already helping to build sufficient herd immunity for the fall months, which will protect healthcare workers from potential overload resulting from new virus variants.

In this interactive simulation, you can learn how the vaccination uptake rate in the population affects the risk of contracting or dying from COVID-19. Although vaccination is no longer as effective in preventing infection with the omicron variant as it was with the delta variant, it is still very effective in protecting against severe disease and death from COVID-19.

Since the actual risk is always highly dependent on the actual pandemic situation, the values are to be understood as estimates.

[interactive risk simulation/population-based]

© 2023 Wegwarth O et al. *JAMA Network Open.*

eTable 1. Risk Ratio for Vaccine-Related Outcomes

**Risk_per_age**	**age_18-34y**	**age_35-59**	**age_60-79**	**age_79≥**
Vaccinated				
Infected	40%	40%	40%	40%
Hospitalized after infection	0.24%	0.72%	3.6%	6.4%
ICU after infection	0.006%	0.017%	0.15%	0.30%
Dead after infection	0.00015%	0.005%	0.15%	0.50%
Side effects	0.02%	0.01%	0.01%	0.01%
Unvaccinated				
Infected	100%	100%	100%	100%
Hospitalized after infection	0.6%	1.8%	6.0%	16.0%
ICU after infection	0.04%	0.11%	1.0%	2.0%
Dead after infection	0.001%	0.03%	1.0%	3.0%
Side effects	0 %	0%	0%	0%

© 2023 Wegwarth O et al. *JAMA Network Open.*
