# Supplementary material for: Vaccination Intention Following Receipt of Vaccine Information Through Interactive Simulation vs Text Among COVID-19 Vaccine–Hesitant Adults During the Omicron Wave in Germany
Source: JAMA Netw Open. 2023 Feb 16;6(2):e2256208. doi: 10.1001/jamanetworkopen.2022.56208 (PMC9936332; doi:10.1001/jamanetworkopen.2022.56208)
Supplement: Supplement 2. — Data Sharing Statement [file jamanetwopen-e2256208-s002.pdf]

Data Sharing Statement

Wegwarth. Vaccination Intention Following Receipt of Vaccine Information Through Interactive Simulation vs Text Among COVID-19 Vaccine-Hesitant Adults During the Omicron Wave in Germany. *JAMA Netw Open*. Published February 16, 2023.
doi:10.1001/jamanetworkopen.2022.56208

Data
Data available: Yes
Data types: Deidentified participant data
How to access data: Open Science Framework (https://osf.io/jrczy/)
When available: With publication

Supporting Documents
Document types: Statistical/analytic code
How to access documents: Open Science Framework (https://osf.io/jrczy/)
When available: With publication

Additional Information
Who can access the data: researchers whose proposed use of the data has been approved
Types of analyses: for a specified purpose
Mechanisms of data availability: with investigator support
Any additional restrictions: none
